# The Lifespan of Human Activity Recognition Systems for Smart Homes

**DOI:** 10.3390/s23187729

**Published:** 2023-09-07

**Authors:** Shruthi K. Hiremath, Thomas Plötz

**Affiliations:** School of Interactive Computing, Georgia Institute of Technology, Atlanta, GA 30308, USA; shiremath9@gatech.edu

**Keywords:** smart homes, lifespan, data-driven procedures, minimal supervision, human activity recognition

## Abstract

With the growing interest in smart home environments and in providing seamless interactions with various smart devices, robust and reliable human activity recognition (HAR) systems are becoming essential. Such systems provide automated assistance to residents or to longitudinally monitor their daily activities for health and well-being assessments, as well as for tracking (long-term) behavior changes. These systems thus contribute towards an understanding of the health and continued well-being of residents. Smart homes are personalized settings where residents engage in everyday activities in their very own idiosyncratic ways. In order to provide a fully functional HAR system that requires minimal supervision, we provide a systematic analysis and a technical definition of the lifespan of activity recognition systems for smart homes. Such a designed lifespan provides for the different phases of building the HAR system, where these different phases are motivated by an application scenario that is typically observed in the home setting. Through the aforementioned phases, we detail the technical solutions that are required to be developed for each phase such that it becomes possible to derive and continuously improve the HAR system through data-driven procedures. The detailed lifespan can be used as a framework for the design of state-of-the-art procedures corresponding to the different phases.

## 1. Introduction

Developing human activity recognition (HAR) systems is at the core of ubiquitous computing systems. With the recent resurgence of interest in smart home environments and with the availability of technology to make smart devices helpful and engaging for their end users [1,2], numerous applications that provide assistance to residents are now available. Owing to the reduced cost of sensors and the advancements in Internet of Things (IoT) technologies, as well as the accessibility of reliable and inexpensive sensing and computing technology, instrumenting homes with sensors for everyday activity recognition in real-world living environments is now a realistic option for many. This has encouraged, for example, the widespread use of pervasive sensing devices in ambient assisted living (AAL) environments. Initial works in AAL were focused on providing care for the elderly population [3,4], but, with the aforementioned advancements, the availability of such systems for diverse populations is now possible. Although systems developed earlier faced technical challenges corresponding to data collection and automated analysis [5,6,7,8], with the present advancements, this is not an issue that current deployments have to encounter. Even when such advancements have made the data collection process seamless and straightforward, substantial challenges remain in developing and deploying HAR systems in smart homes.

“Off-the-shelf” human activity recognition systems are desirable in smart homes, since they promise to be deployable “as-is” and immediately into any environment while not requiring additional resources in terms of cost, effort, or time. However, given the individualized settings and the idiosyncratic behaviors of residents, it is not realistic to assume that such systems will work without the need for adaptation to such individualistic environments. Prior work shows that these adaptive procedures require experts in the loop to identify sensor grouping based on the location, function, and mapping of similar types of sensors between the source and target domains, without guarantees of obtaining optimal performance in the target environment [9,10,11]. Additionally, the goal to provide a fully functional system, requiring sample-precise annotations and the development of tailored HAR systems, that cater to specific home settings and individuals is realistic.

In this “perspectives” article, we define the lifespan of a HAR system for smart homes, i.e., we conceptualize how such “bespoke” systems can be derived for a range of practical application scenarios of sensor-based human activity recognition in smart homes. We do so by analyzing relevant related work and contextualizing the derived concept—the “lifespan”—with respect to realistic application scenarios. This addresses the concerns arising from the unavailability of an “off-the-shelf” system, which is often hard to achieve. Thus we accomplish the following: (i) discuss the challenges in developing HAR systems for smart homes and state-of-the-art procedures; (ii) detail a typical application scenario in the smart home, which serves as a guideline for detailing the lifespan of HAR system; and (iii) provide the technical details of the different components that make up the lifespan of the HAR system.

## 2. Background

With decreasing sensor costs, automating ‘regular’ homes has become a possibility for many. IoT or environmental sensors can collect data for extended periods of time without concerns regarding battery recharging or privacy. Such sensors capture data on the detecting motion [12] or on the interaction with objects that are instrumented with sensing capabilities [13]. As such, these advances have enabled the recognition of the activities of daily living for ambient assisted living (AAL) environments. Such automated assessment systems are required for home automation [14].

### 2.1. Smart Homes

Mark Weiser, in his seminal paper ‘The Computer for the 21st Century’, stated

“The most profound technologies are those that disappear. They weave themselves into the fabric of everyday life until they are indistinguishable from it.” [15]

Toward this goal, researchers in ubiquitous computing aim to enable and provide computing away from the desktop. A unifying research theme that arose out of this was to focus on the computing needs in everyday lives, especially those that were away from work or office spaces. This initiated the research effort towards investigating computing in the home [16]. Smart Homes are a branch of ubiquitous computing that involve incorporating “intelligence” into living spaces. Smart homes for health care have been described as “a home equipped with smart sensors such as Bluetooth, Wi-Fi, or similar technology, not restricted to IoT, to automate, regulate, and monitor home occupants’ physical health, mental health, and environments within the home. The focus must be on convenience, safety, and improvement of one’s quality of life, to address the needs of the individuals, caregivers, and health professionals” [17]. Through ambient intelligence systems, the goal is to monitor smart homes and provide the control of home appliances and devices to users such that it enables them to execute tasks automatically. Ambient intelligence provides for instrumented environments that are sensitive and responsive to the presence of people [18] through providing intelligent monitoring and access control [19].

The services facilitated through automating homes can be broadly classified into three major categories: (i) Comfort; (ii) Healthcare; and (iii) Security [19]. Through the provision of comfort, smart homes provide ease in daily life. “Optimization for comfort in the environment is possible through the identification and automation procedure, learning user behavior, tracking user location, identifying the user, and automating tasks. Remote access and control enable users to remotely access, monitor and control their home environment” [19].

Smart homes also support providing healthcare for their inhabitants. As stated in [19], a significant portion of the world’s population would be considered the elderly by the year 2050. To aid such elderly individuals in living independently, maintaining the safety of such individuals is of paramount importance, which can be achieved by detecting and preventing accidents, such as detecting an event such as a fall and calling for emergency services when such an accident occurs. Supporting aging in place (also termed as ‘Gerontechnology’ [20]) is another important aspect of helping senior adults with daily living activities, such as reminding them to take medications. One such research initiative at Georgia Tech devoted to the multidisciplinary exploration of emerging technologies and services based in the home is that of the Aware Home Research Initiative (AHRI). Supporting busy families in the generation where adults work full-time jobs is another scenario where smart home automation provides support. Often times, such families will have both elderly parents as well as young children that they must care for and thus the term coined for such populations is the “Sandwich Generation” [1].

Research into smart homes gained interest in academic domains, where the focus was on providing context awareness and smart decision making in automated environments. A number of initiatives have been focused on providing the following: (i) home automation, where providing comfort and convenience are of importance along with the goal of saving energy and resources; (ii) facilitating safety and security, where the goal is to provide monitoring of the inhabitants’ well-being and aim for the provision of safety and security such as burglary recognition; and (iii) for entertainment, where the goal is to connect users and media with each other and to facilitate communication. A number of research works [1,2] were introduced with the goal of providing such services. One of the major applications was to provide assistance and support to the elderly to make their lives easier and provide support in daily living activities.

Toward the goal of automating homes to make human lives easier and more comfortable, automation efforts were initiated as early as the 19th century [21], where home appliances were designed to automate chores. Echo IV [22] was the first smart device that was used to manage shopping lists, control the home temperature and humidity, and provide tips for cooking. With technological advancements such as microcontrollers [23] and transistors [24], as well as reductions in the costs required for instrumenting homes with sensors, new application fields of computing—such as smart home automation—have become a possibility.

A few of the smart home automation research developed were the MavHome [8], GatorTech [2], and the Ambient Kitchen project [25]. MavHome [8] was set up with the goal for the environment to understand the resident’s activities and respond accordingly to assist them in their daily living routines. GatorTech [2] (an extension of the Matilda Smart Home [26]) created an actual live-in environment aimed at assisting older individuals and those with special needs. Similarly, the Ambient Kitchen project [25] was aimed at developing a high-fidelity prototype by instrumenting objects used in the home to design applications that assist in everyday environments.

During the early 2000’s, there was a push from the industry towards automating smart homes, and a number of appliances were developed that aided in this automation process. This was possible due to the advent of technology that aimed at making smart homes more accessible, engaging, and helpful to their end users [27,28,29]. With recent advancements in IoT-based technologies and cloud computing practices, there has been a renewed interest in research and development efforts. The focus has also now shifted toward private home automation. With the increasing number of smart home devices and vendors, sophisticated industry standards and platforms, and the number of assistants embedded in the environment, interest in developing smart home-based devices is evident.

Designing HAR systems has been made possible through the availability of data repositories such as the CASAS smart home datasets [30] and virtual smart home simulators such as the Home I/O simulator [31] or the VirtualHome simulator [32,33,34,35]. Smart homes in [30] are aimed at identifying analysis procedures that aid in discovering user patterns. With the advances made in machine learning and data analysis techniques, it has become possible to analyze the data collected in such environments [36].

### 2.2. Activity Recognition in Smart Homes

Human activity recognition (HAR) is aimed at identifying activities that are performed by a person as a result of analyzing the data collected from various sensing mechanisms [37]. Equipped with sensors and actuators, smart homes aim to not only detect movement within the home [38], but also to identify interaction with objects, devices, and appliances. All such devices, sensors, actuators, appliances, and objects in the home are interconnected through communication protocols [39].

To utilize the services provided through the smart home, it is essential for the home to understand and recognize the activities of the residents. Activity recognition systems in smart homes are typically designed and deployed to provide such recognition capabilities. Through the process of logging identified daily living activities, changes in regular routines can be indicative of health-related concerns that can be used to inform residents and their caregivers [6,7,30,40,41,42].

Sensing modalities that are used to detect movement in smart homes primarily belong to either vision-based or sensor-based systems. Vision-based approaches use perception-based sensing mechanisms, also known as optical sensors, to capture data for analysis [43,44]. These optical sensors aid in the collection of 2D images, 3D images, and video data. The use of depth-video-based HAR designed for elderly health care monitoring utilizes skeleton joint features to analyze behaviors and their changes therein [45], and a depth-silhouette-based human activity recognition system has been used for the real-time logging of performed activities [46]. However, this sensing modality comes with privacy concerns, where residents may not be willing to accept the information collected through the vision-based sensing mechanisms [47,48].

Sensor-based HAR systems comprise on-body or wearable sensing [49], sensors placed on objects [25,50], and ambient or in-the-environment sensors [51]. HAR methods comprise various sensors that are networked and connected with numerous devices to track the resident’s activity or behavior. Since these modalities either record data through state changes (ambient sensors) or more continuous-valued data (wearables), the data recorded provides for a time series analysis problem. The sensor-based HAR system is less privacy intrusive and has thus been widely accepted to monitor the activities of daily living [52,53].

#### 2.2.1. Human Activity Recognition Systems

The data collected in smart homes is obtained through recording the values of sensors that are used to capture the way residents interact with their respective environments. Recordings that capture the actions and interactions of the resident are then used to analyze the activities of daily living of the resident [39,43,48]. Analysis procedures either make use of contextual knowledge such as the location, time and frequency of activities, spatiotemporal information, and interactions of the residents with objects [39,54]. Such procedures have been termed as ‘Knowledge-Driven Approaches’ and require knowledge from domain experts to design the system [39]. Ontology-based approaches are used to build these context-aware applications [55]. Complementary to this kind of analysis is ‘Data-Driven Approaches’, where statistical models are built using the data recorded in the smart home. These require large amounts of annotated data to learn probabilistic machine learning models, such as hidden Markov models, K-nearest neighbors, etc., that can recognize the activities of residents [39,56,57]. Although, both ‘Knowledge-Driven Approaches’ and ‘Data-Driven Approaches’ can be used to learn activity models, due to the individualistic environments of smart homes that require knowledge of the idiosyncratic behaviors or residents, either complete knowledge of the environment or large amounts of annotated data would be required for building activity models [54]. This either requires domain expertise or large amounts of wait time (to collect annotated data) for an activity model to be available to residents.

Since ambient sensors record state changes, the time series data recorded results in an irregular sampling rate, which is unlike the data recorded from wearables or videos, where data collected from these modalities have a more consistent sampling rate. In order to identify the points in time where behavior changes occur in the time series analysis problem, algorithms belonging to the family of change point detection (CPD) methods are used [58]. A number of CPD-based methods have been applied to segment time series data into activities of interest—also known as ‘Activity Segmentation’. Algorithms are developed to identify the activity segments automatically and then the activity in those segments is identified through a recognition procedure [59]. Real-time activity recognition can be essential when identifying activities that require immediate care such as fall detection [60] or to automatically log behaviors for health monitoring. Some CPD methods that have been developed are suitable to provide real-time activity recognition systems, whereas others recognize activity segments after a delay from the time of occurrence. Different windowing procedures have been compared in [61] to estimate activity boundaries. Identifying explicit windows (EW) corresponds to the ‘pre-segment’ technique [62,63] where a given window contains all the sensor events corresponding to a given activity. Annotations are required to identify the beginning and end points of the activities of interest. When no such annotations are available, change points corresponding to when changes occur in activities are identified using the statistical and probability-based measures described in [64,65]. In order to perform activity recognition, it becomes essential to identify all segments corresponding to the activity instances, since the whole segment is used to predict a given activity. Thus, it is not straightforward to use the ‘pre-segment’ technique in real-time analysis. For real-time analysis, the time window (TW)—where windows span over a specific duration of time [66,67,68,69,70]—the sensor event window (SEW)—where windows span over a specific number of sensor event triggers [10,71]—has been used. Although, the time-based windowing technique is favorable for regularly or continuously sampled data over time when commonly used with wearable sensors, the data collected through ambient sensors can be sampled at regular intervals through a forward-filling procedure. In both cases, however, identifying the ideal window length of the sliding window can be challenging and requires domain-specific knowledge to estimate the window length [72]. Heuristic approaches such as rules, thresholds, and dissimilarity measures between window embeddings have been used to identify window lengths dynamically [67,73,74].

A number of traditional approaches have been proposed for sensor-based HAR systems in smart homes. Classification approaches such as random forests, naive Bayes, decision trees, and conditional random fields have been explored [75]. Some of the traditional feature representations look into the number of sensor firings, the time spent at a given location, and the time spent moving between locations [76]. Another work made use of the features that model contextual information by considering the mutual information between sensor events and decay in sensor event triggers [10]. Spatiotemporal features explored with multilayer perceptron, hidden Markov models, decision trees, etc. have been shown to outperform traditionally used feature representations [77]. The use of SVMs and variants of incremental SVMs have been employed to improve the performance of the HAR systems [78]. In [79], the authors made use of sensor data contribution significance analysis and spatial distance matrices to identify (a) the relevant sensors that are most informative of the activities in the home; and (b) the noise caused by various factors such as pets and visitors that are not relevant to the activity being monitored. Eliminating the noise and sensors that do not contribute to activity recognition has been shown to improve performance scores. Inspired from the cluster then classify paradigm, in [4], the location was used as the contextual information to cluster data points that were assumed to belong to the same activity. Some of these conventional procedures require handcrafted feature extraction methods to learn relevant information from the sensor event triggers [39] or require large amounts of labeled data to build knowledge [79]. Recent work [80] looked at different metrics that could be employed to obtain features that are of relevance for classification. This still requires domain knowledge to initialize the entire list of features that are used as a starting point. A subset of these features is then deemed to be important for the classification procedure. Similar drawbacks exist in activity recognition systems that make use of ontologies where detailed information about activity interactions is required for generating feature vectors [55].

With the advancements in deep learning techniques, it has become possible to model high-level abstractions from complex data [81]. Deep learning techniques can be used to learn good high-level feature representations from raw signals by utilizing unsupervised learning procedures, without requiring any manual engineering efforts. Such procedures can also be used for end-to-end learning systems where the models perform classification using the automatically extracted features [82,83]. Convolutional neural networks (CNNs) have been used to capture local dependencies in time series data. Since they are invariant to scale and translation, they are able to capture the local temporal dynamics between data points [84]. Deep convolutional neural networks have been used to analyze sequences of binary sensors that are converted into gray-scale images [85,86]. A 1D-CNN structure developed on raw data sequences was used in [87] to extract high-level features, to learn mappings between sensor event triggers, and for activities in an end-to-end learning procedure. To capture the temporal information in sequential data modeling procedures, RNNs are employed. LSTM networks have become popular due to their ability to capture long-term dependencies [84]. Thus, different variants of LSTMs such as bidirectional and cascading LSTMs have been used to automatically learn temporal information from raw sensor sequences and achieve reasonable performance outcomes [88]. Using frequency encodings to capture the co-occurrence of sensor event triggers in a sequence has been used to model and learn good feature presentations that are then passed through a fully convolutional network [89]. Other embedding procedures inspired by language-based modeling techniques such as ELMO have been used to learn better feature representations [90]. Activity2Vec [11] is a sequence-to-sequence model that is aimed at learning feature representations for the activities of daily living and activities that occur rarely such as fall detection. Although most of these deep-learning-based approaches achieved state-of-the-art performances, they have a major drawback—they make use of ‘pre-segmented’ activity instances as inputs to the classification procedures. The use of the pre-segmentation technique to identify explicit windows (EWs) is not ideal, since systems that require knowledge of the start and end points of activities cannot be used in the deployment scenario.

More recently, graph attention networks have been used as a classification approach due to their increasing prevalence in various fields [91]. By utilizing a graph structure to model human activities, a sequence is converted into a sequence graph, where each graph node is connected to one or more nodes [92]. Information is aggregated through messages received from neighboring nodes in the graph and is then transmitted. An attention mechanism is used to produce node representations that allow for distinguishing the contribution of each node on the target node. The work in [93] used sensor events as nodes, and the edges between the nodes were represented by the intensity of the connections, thus forming a graph. The graph attention network proposed in this work makes use of the generated features to learn a location-oriented and time-oriented graph attention network that is further passed through convolutional layers and subsequently through a fully connected layer to identify the activity performed.

#### 2.2.2. Active Learning

The successful implementation of a human activity recognition system requires the sensor data gathered in the smart home to be accurately mapped to human behavior. Developing supervised training approaches requires large amounts of annotated data to achieve reasonable performance scores [94]. An annotation procedure is employed to provide labels to activities that are identified over the duration of data collection in the homes. A significant challenge in obtaining a large number of such annotations is that it is cumbersome and requires time and effort [95]. Annotator expertise determines the quality of the annotations obtained, which can be expensive based on the expertise of the annotator. Annotations can be obtained either in situ by residents while the activity is being performed or retrospectively, wherein either the resident or an external annotator provides the annotations by observing the data collected. Obtaining such labels could cause disruptions by interrupting users frequently or requiring too much effort and time when providing such labels retrospectively. As the recognition system evolves, new sets of activities may be picked up by residents, such as picking up dancing as a hobby, or the initially recognized set of activities can be performed differently, such as relaxing as an activity that could change in definition from reading a book to taking a nap. Thus capturing activities of interest and variants of these, without burdening the resident, requires the use of a semisupervised machine learning paradigm—active learning.

In contrast to supervised approaches that require annotations for all the collected data, active learning (AL) queries only those data points that are deemed informative to the learning procedure. As such, two different components for identifying such data points exist—(i) sampling strategies and (ii) query strategies. Sampling strategies determine the procedure by which the data points to be picked for the annotation procedure are identified. Two popular sampling strategies corresponding to pool-based active learning and stream-based active learning are used [96,97]. In the pool-based active learning procedure, which is suitable for an offline learning procedure, a large unlabeled dataset and a small labeled dataset are utilized. The algorithm then selects the best data points that are most informative from the unlabeled dataset to be queried. Similarly, in the stream-based active learning procedure, which is suitable for an online learning procedure, streams of data are analyzed for obtaining an annotation based on the informativeness of the incoming data point. Query strategies determine the specific data points to be identified based on the informativeness of the data points for the learning procedure. Two popular query strategies correspond to the uncertainty sampling and the margin-based uncertainty sampling, where, for the first strategy, the model queries for those data points that it is least confident about [98]. In the margin-based uncertainty sampling, the model queries for those samples for which the margin between the two most probable class prediction probabilities is small. Other query strategies such as query by committee [98] and logistic margin sampling [99] have been explored. To determine the number of data points to be queried for a budget, this process is predetermined. The size of the budget determines the performance gains that can be achieved and how close the AL procedure reaches the performance measures corresponding to supervised methods. Various strategies for how this determined budget is spent in obtaining the annotations are discussed in [100]. Active learning procedures have been utilized in smart home settings to learn about resident activities and behaviors, wherein they use the sampling and query procedures described in [101]. Markov decision processes that make use of gestures and vocal expressions to obtain feedback from the resident, which are incorporated into the modeling procedure. Positive and negative responses to their interactions with the system are provided as feedback to the modeling procedure [102]. Other works have used contextual information such as location to annotations corresponding to informative clusters [4,103].

## 3. Need for Bespoke HAR Systems in Smart Homes

By critically analyzing the state-of-the-art approaches developed for activity recognition in smart homes, we discuss the drawbacks of said systems. Although the recognition scores obtained through systems in [88,89,90] wer high, such systems are not ideal for providing an HAR solution, since they require event-based sequences, where knowledge of the start and end times corresponding to a given activity sequence is needed. These systems, when deployed in smart home scenarios, require the resident to provide information regarding this, which is often cumbersome. The resident will have to either be continuously engaged with the system to provide these activity boundaries or provide this information retrospectively, which may be subject to recall bias [104]. Thus, techniques developed for the ‘pre-segmented’ activity instances are not readily deployable in real-world scenarios. Although the recent literature makes use of robust sequential models such as LSTMs and graph attention networks, the reliance on the identification of ‘pre-segments’ limits the deployable capabilities of the systems [88,90]. Some such systems are listed in Table 1, which correspond to the category of ‘Event-based analysis’. As observed from the literature, most other procedures to identify the appropriate data sequence length for analysis require some knowledge of the activities performed by the resident [10,66,67,68,69,70,71]. Other approaches require the procedure to be context-aware (e.g., location) through incorporating knowledge of the environment [93]. This requires domain expertise to build such knowledge for every environment analyzed. Examples of such systems are listed in Table 1, which correspond to the category of ‘Requires domain knowledge’.

The methods developed in [11] have been tested on some of the collected CASAS datasets but not on others; thus, it is not clear if the given method generalizes across different smart home settings. The works in [8,9] developed methods for specific homes [5] by making use of object and appliance interactions (developed through ontologies) that do not generalize to other smart homes, which consist of different objects. Differences in the sensor positions in different home settings lead to changes in sequence patterns, which were utilized to model activities in [8]. This is a typical issue with some of the related work. Similarly, the works in [8,9] developed systems that are specific to the given smart home of PlaceLab [5] and MavHome [8], respectively. Contextual information, which is specific to individualized settings, has been utilized in developing ontologies [105] and in building recognition procedures that require context-sensitive embeddings (the examples in Table 1 corresponding to the category of ‘Requires domain knowledge’) [106,107,108].

The HAR system developed in [30] used specific information in the home for feature engineering such as the number of sensor events triggered, recent sensor events, and contextual information such as the day, week, or the hour of the day, as well as information related to the resident’s activity patterns such as the elapsed time for each sensor event. These details change across different smart home settings, and, thus, the HAR system would require such details for every individual environment. As seen in [11], the developed sequence-to-sequence model was developed for a given smart home of the CASAS dataset—HH101 [30]. Similarly, in [109], the activity boundaries were identified using statistical measures such as the Pearson coefficient, but it is unclear if it is applicable to smart homes that are more complex. Such handcrafted features require large amounts of annotated data, which are required to be collected in the smart home [94] to learn representations that are suitable for a given home. Some such systems have been listed in Table 1, which correspond to the category of ‘Requires annotated data’. These would lead to building robust supervised learning procedures. As discussed, such analysis procedures require large amounts of resources in terms of annotations, time, and effort [95], and the developed system becomes available only after long periods of wait time (required to collect data in the given home), which might not be acceptable to the resident [110].

To summarize, the design of HAR systems for the application scenario of smart homes is challenging and not straightforward. Existing HAR systems cannot be used “as-is” when deploying them to a new smart home setting, and each deployment requires domain-specific modifications in order to be usable and effective. Detailed knowledge of the target environment such as activities performed in the home, extensive ground truth annotations for such activities, and precise activity boundaries are required for building most state-of-the-art HAR techniques in smart home scenarios. Such knowledge is obtained in the form of detailed extensive ground truth annotation and frame-precise activity segmentation. Thus, developing a supervised HAR system for specific settings requires large amounts of annotated data. Obtaining such annotations from residents is burdensome and requires considerable effort [98,99]. We identify and list the drawbacks of the aforementioned HAR systems as follows: (i) most systems are not developed for providing automated recognition; (ii) idiosyncratic environments require the development of HAR systems that are specific to the settings of smart homes; and (iii) the approaches developed for a given smart home environment do not necessarily generalize to other smart homes. Hence, “off-the-shelf” systems that will be universally useful without tuning them for individual smart homes do not exist. To address the mentioned concerns, we motivate the development of bespoke HAR systems for such individualized settings that learn from scratch in a given environment, with minimal involvement from the residents. Such a system is developed through a data-driven procedure and does not make use of specific (contextual) information in a given setting.

We designed bespoke HAR systems for these individual environments by defining the various components that they should encompass, where the goal was to develop a fully functional HAR system. Such a system was derived through the assumption of a cold start scenario, where (initially) unlabeled sensor data were passively observed in the smart home. Thus, the design of the system did not require any contextual information or knowledge specific to a given home setting. An initial functional HAR system becomes quickly available to the resident without requiring long periods of wait time. As the system observed data in the home, it built and incorporated knowledge hierarchically. Minimal supervision provided by the residents was used to develop these HAR systems and their updates therein in order to reduce the effort and burden on the resident. To motivate the technical aspects of the said lifespan, we detailed an application scenario that observed the different scenarios that are typical in a smart home. Technical aspects were designed through various components to address these different scenarios in the home.

## 4. The Lifespan of Human Activity Recognition Systems for Smart Homes

**Figure 1:**
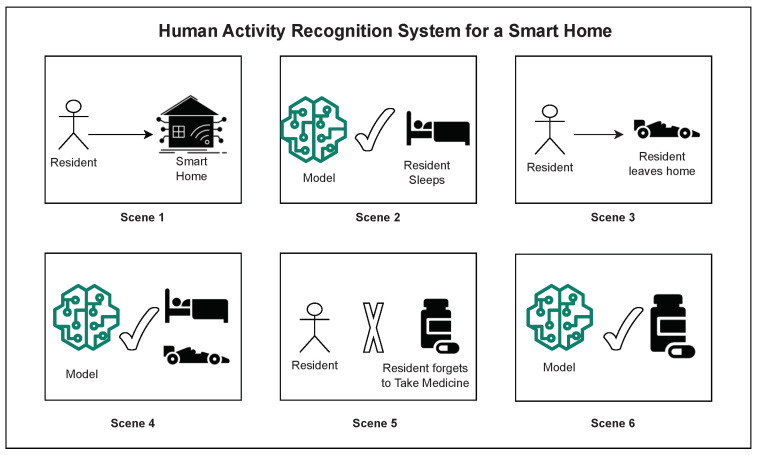
The lifespan of a human activity recognition system for smart homes in terms of requirements evolving over time and the needed responses of such systems to a life that is ever-changing. See text for description.

To motivate the need for defining the lifespan of HAR systems for smart homes, we provide an illustration of a typical application scenario for activity recognition in these settings, which is shown in Figure 1. It details the scenario in a smart home and the need for a functional HAR system that addresses these. In the beginning (Scene 1), a resident moves into a new smart home, with installed motion and door sensors to track movement patterns that are essential for activity monitoring. Given that no actual HAR system exists at this time (“cold-start”), because “off-the-shelf” HAR systems will not work in highly individualized and situated environments such as a private home.

As such, an initial bootstrapping procedure was deployed that first collected raw sensor readings while the resident conducted their regular activities (Scene 2). The initial HAR system aimed at detecting the most prominent or frequently occurring activities in the home (e.g., ‘sleeping’, as shown in the figure). However, the resident also performs less-frequent activities such as ‘leaving home’ (Scene 3), which do not get modeled yet. ’Leaving Home’ can be considered as a less-frequent activity in the case of an elderly living environment, where the activity is not one of the prominent activities. The HAR system would now be continuously updated to capture both the more and less prominent activities in the smart home (Scene 4).

As the resident continues to live in the smart home, the model additionally focuses on assessing activity routines. Most developed HAR systems are utilized to identify activities that help with logging behaviors performed by residents. However, there is more to understanding these behaviors than just recognizing individual instances. Thus, the assessment of routines helps in the analysis of (any) changes that occur in a resident’s life at a level higher than just analyzing individual activity patterns, for example, when the resident is forgetting to “take medicine” (Scene 5). Identifying *regular* routines can inform any deviations that will be used to inform the resident or caregivers (Scene 6).

In order to detail the technical concepts that aim at capturing the scenarios illustrated in Figure 1, we provide detailed technical specifications of the various components of this overarching technical concept in what follows. The development of individualized HAR systems for smart homes requires minimal human intervention, and the focus is on the rapid availability of essential functionality to the resident. As part of the technical details, we also ensure that the HAR system continuously improves and adapts to changes in the home system over time.

### 4.1. Phase 1: Bootstrapping

**Figure 2:**
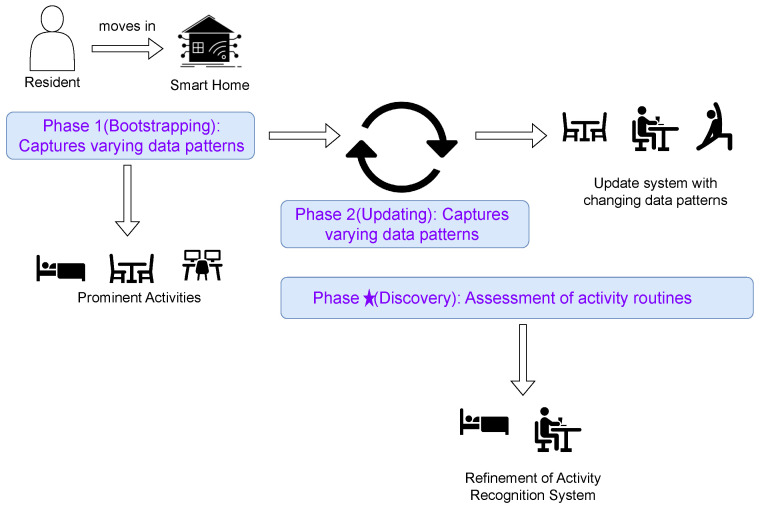
Illustration of the three components of HAR system for smart homes consisting of *bootstrapping, updating, and assessment*. Phase 1: An initial, fully functional HAR system is bootstrapped from scratch in a data-driven procedure. Phase 2: Maintenance of the sensor-based HAR system. Phase ★: Aims at the assessment of activity routines.

Figure 2 illustrates the various components of the HAR system as we defined it to provide the basis for the design of the lifespan of an HAR system for smart homes. At the beginning of the HAR system (Phase 1 in Figure 2) there is a bootstrapping procedure, which aims at getting the first working version of the HAR system in place, thereby focusing on minimal user involvement and rapid deployment. As such, it collects unlabeled sensor readings as soon as the resident moves into the home, thereby targeting the most frequently occurring and most prominent activities in a specific home. Identifying such prominent activities, albeit certainly not all of them, has more practical value than designing a sophisticated system that would require large training data and a longer wait time for the resident, thus, overall requiring more resident involvement. Residents are expected to be involved at a minimal level primarily to confirm the identified sequences of movement patterns, which correspond to the aforementioned most frequent and prominent activities, which will be modeled by the HAR system. The resident is asked to provide annotations in an active learning scenario [98]. Annotations corresponding to only the most prominent movement patterns are requested from the resident. These patterns can be identified through a set of designed filtering procedures that are implemented during the construction of the initial analysis pipeline.

The design of the state-of-the-art analysis pipelines faces technical challenges due to the sparse set of annotations obtained from the smart homes provided by residents. Additionally, there is a lack of availability of high-quality annotations, since these annotations come from the resident, who provides them retrospectively. Thus, the goal of this stage, as described in previous work, is “to provide a system that “jump-starts” the activity recognition pipeline for the smart home” [111].

At the end of this initial phase, the HAR system should be able to recognize the most prominent activities with satisfactory accuracy for a subset of activity classes observed in the smart home. Also, there is typically more happening in a home than those limited sets of activities, which corresponds to those activities that are not captured by the initial system and variants of the initial set of captured activity classes that are missed [112,113]. However, with such an automatically derived (“bootstrapped”), functional HAR system, the smart home can already fulfill a range of routine operations. For example, it would be able to monitor and track sleeping patterns or the work-life balance of its residents. This phase corresponds to Scenes 1 and 2 in Figure 1. Previous work has presented prototypes that cover Phase 1 of the lifespan of a smart home’s HAR system as we have defined in this paper (e.g., [111]).

### 4.2. Phase 2: Updating

In the next phase of the HAR system (Phase 2 in Figure 2), an initial HAR system (from Phase 1) will be updated and extended in an incremental fashion—as more data is captured in the smart home, this likely increases the variability of the activities themselves, as well as their individual appearances. At the technical level, updating and extending an initial HAR system is targeted by the second phase of the lifespan, and it requires a *class-* as well as *style–incremental* approach. Through the former, the HAR system learns to identify new activities, and through the latter, the HAR system learns to identify variants of already identified activities, and, as such, refines them with regard to, for example, more accurate segmentation and/ or covering specific variants of individualized activities. An example corresponding to a new class corresponds to an activity that the HAR system cannot recognize at the end of Phase 1, for example, ‘leaving home’, which occurs infrequently as shown in Figure 1. As the resident continues to stay at home, they may adopt different ways to relax, which, for example, may change from reading a book to taking a nap. This corresponds to identifying changes in activities already identified through Phase 1. This phase corresponds to Scenes 3 and 4 in Figure 1.

Building on the initial HAR system (from Phase 1) shifts the manual efforts, which are still required for the occasional annotation of significant, new movement patterns, which are away from the activity patterns that are already known (from Phase 1) and thus keeps the burden on the resident at a reasonable level. A suitable approach to accommodating novel activity class instances and potential concept drift in already modeled activities is through utilizing continual learning (CL) models [114,115,116,117,118,119,120] when such instances are observed. Continual learning as a concept corresponds to situations when a model—any model not restricted to the recent surge in CL research in the deep learning community, but rather which adopts the general concept of continuously updating existing HAR models to changed circumstances—learns sequentially from data or tasks without forgetting knowledge obtained from preceding tasks. Throughout Phase 2—which, essentially spans from the end of Phase 1,i.e., when the first HAR system is available—the HAR system is continuously updated and extended with the goal of keeping up with the ever-changing circumstances of life, which, however, does not forget the already learned concepts (activities and their styles).

### 4.3. Phase *★*: Routine Discovery

Phases 1 and 2 aim at developing a functional HAR system that recognizes regular activities in the home. To complete the lifespan of the HAR, we next detail the final phase to consist in assessing activity routines. Thus, Phase ★ (occurring in parallel to Phase 2) is aimed at using the developed activity recognition system in assessing these routines. Monitoring these routines helps in establishing regularity (or not) of the behavior patterns of the resident. Thus, this phase is aimed at identifying these different routines in the home.

The previously described phases detailed the recognition system that can be utilized for monitoring the activities of the resident. In addition to providing such monitoring, assessing the activity routines of the residents is beneficial in the long-term goal of providing assistance in smart homes, for example, in providing care to the resident. Activity routines are patterns of behavior constituting sequences of activities [121]. Monitoring the regularity of such routine patterns can inform of the resident’s health and be utilized in, for example, observing circadian rhythms [122]. Aberrations from *regular* routines are indicative of deviant behaviors which may be a cause for concern. Hence, changes in behavior patterns can be informed to caregivers, especially in the case of the elderly. Thus, this phase—Phase ★ of the lifespan of the HAR (Figure 2)—is aimed at assessing activity routines from the recognition models identified previously. Refinement of the identified activity routines can be further used in refining the designed recognition system through the previous phases. This phase corresponds to Scenes 5 and 6 in Figure 1.

## 5. Scalability of the Proposed Conceptual System

Developing an activity recognition system for the settings of smart homes is not straightforward. Several challenges to developing HAR systems for providing activity monitoring in smart homes exist. Most of the developed state-of-the-art procedures are not aimed at providing automated recognition, and they often-times make use of ‘pre-segmented’ activity instanced for analysis. Smart homes are individualized environments with the idiosyncratic behaviors of residents. Thus, HAR systems are required to be developed specifically for the settings of a given home. This leads to a lack of an “off-the-shelf” HAR system that can be used “as-is” in a different smart home setting. The lack of such universally usable systems, which require at least some modifications in terms of tuning to different smart homes, motivates the need to develop “bespoke” HAR systems for such environments.

To aid in developing the “bespoke” HAR system, in this work, we propose a conceptual system. This conceptual system details the lifespan of a HAR system for smart homes. The designed lifespan provides for the different phases of building the fully-functional HAR system, which require minimal resident supervision. Such a designed lifespan provides for the different phases of building the system, which is motivated by a typical application scenario in the home setting. Through the use of data-driven procedures, the technical solutions developed for each of these phases makes it possible to derive and continuously improve the HAR system. We discuss the scalability of the proposed conceptual system with regard to (i) the performance in new and unseen environments and (ii) the performance in multi-resident smart homes.

Developing machine learning models on a generic population, where data is collected from various data sources, and fine-tuning these models to specific populations or individuals is of interest [123,124,125]. At its core, this idea of personalization is aimed at adapting to specific persons to provide for better activity monitoring. In order to achieve this goal, in a fine-tuning procedure, a small portion of data from the specific population or individual is used to fine-tune a model built using generic data. The expectation of building such a personalized model is to adapt to the given specific population. The phases corresponding to Phase 1 and Phase 2 of the conceptual system—the lifespan of the HAR—mirror this phenomenon. In Phase 1, the procedure starts in a cold-start scenario, to “jump-start” the activity recognition system. Since such a system learns from passively observing data in a given home and builds knowledge hierarchically, thus, the procedure is designed to learn the activity patterns of any given setting. Through the update and maintenance procedure, in Phase 2, the initial HAR system is updated through a continual learning procedure. This HAR system learns a refined model that is personalized to a given home setting and the idiosyncrasies of the resident occupying the home. Thus, the conceptual system is a generic approach and can be used “as-is” in any new and unseen smart home. The HAR system developed through the phases of the conceptual system is personalized to the individual home and to the behaviors of its residents.

The design of the conceptual system, with its various phases, does not differentiate between a single-resident household and a multi-resident household. Differentiating between the activities performed by different inhabitants in a multi-resident household is possible using a person identifier, which helps distinguish between the different residents. Various studies have looked into such identification techniques such as, but not limited to, radio frequency signals, WiFi, and using videos. Fusing data from different modalities to analyze activities pertaining to a given individual has also been developed [126]. In the absence of a way to identify different residents, the HAR system learns of the activity patterns in the home without making a distinction between who performed a given activity. However, this is not a limitation of the conceptual system, and these methods can be incorporated into the HAR system obtained from the implementation of the conceptual system. Such integration will result in activity monitoring that learns activity patterns for the different residents in the home.

## 6. Conclusions

A number of activity recognition systems have been developed for smart homes. Analyzing these systems developed across the various smart home datasets [5,8,30] provides insights into the complex and challenging application scenario, which requires such systems to be developed for specific environments [4,8,11].

The proposed lifespan for the HAR is aimed at capturing three important and essential technical challenges that building recognition systems in smart homes face: (i) requiring large amounts of in situ data to build (supervised) activity recognition systems, (ii) requiring a large number of annotations from residents or experts over the entire duration of data collected, to provide labels, and; (iii) requiring a substantial amount of wait time (by the resident) before the system becomes available for use in the home.

In order to primarily address these challenges as observed with the current state-of-the-art procedures, in this “perspectives” track we introduced the lifespan of the HAR systems for smart homes. The lifespan of the HAR addresses the aforementioned challenges by providing a fully functional HAR system that becomes available quickly to its residents—after observing data for an initial period of time and obtaining annotations for the most prominent movement patterns during Phase 1. The update procedure detailed in Phase 2 serves to identify movement patterns that are not observed initially yet require minimal supervision from the resident. Since updates to the system are available after the update procedure, the “new” HAR system becomes available as soon as it is ready for resident usage. Lastly, this developed recognition procedure serves as a tool to understand user activity patterns and regularities in *daily* routines.

## Figures and Tables

**Table 1:** Activity recognition systems developed for smart homes.

Reference	Category of HAR System	Activity Recognition System	Datasets(s)
A sequential deep learning application for recognizing human activities [88]	Event-based analysis; requires annotated data	Variations in sequential modeling techniques (LSTMs) were used on event-based data instances	CASAS datasets (Milan, Cairo, Kyoto7, Kyoto8, and Kyoto11) [30]
Fully convolutional network bootstrapped by word encoding and embedding for activity recognition in smart homes [89]	Event-based analysis; requires annotated data	A word2vec encoding was applied to sensor event-based windows, which were then passed through a fully convolutional network for classification	CASAS datasets (Aruba and Milan) [30]
Using the language model to bootstrap human activity recognition that utilized ambient sensors Based in smart homes [90]	Event-based analysis; requires annotated data	Different embedding techniques were used to obtain the learned features followed by a sequential modeling procedure (LSTM) on the event-based data instances	CASAS datasets (Aruba, Milan, and Cairo) [30]
Activity2vec: Learning adl embeddings from sensor data with a sequence-to-sequence model [11]	Requires annotated data	A sequence-to-sequence model was used to generate features followed by a random forest model for classification	CASAS dataset (HH101) [30]
Enhancing activity recognition using CPD-based activity segmentation [71]	Requires annotated data	A heuristic function followed by a dissimilarity-based approach were used to identify change points. Handcrafted features were extracted. A random-forest-based modeling procedure was employed to perform the classification	CASAS dataset (Apt 101-130) [30]
Using ontologies in case-based activity [9]	Requires domain knowledge	Rules case-based reasoning, where the information gained for each feature for a given activity is used to provide the predictions	PlaceLab [5]
Activity recognition on streaming sensor data [10]	Requires annotated data	Handcrafted features generated over sliding windows. An SVM-based classification model was employed.	Smart home TestBeds—B1, B2, and B3 [62]
MavHome: An agent-based smart home [8]	Requires domain knowledge	Episode discovery algorithm that identified significant episodes in the sequence of patterns mined	MavHome [8]

## Data Availability

Not applicable.
